# Mapping flowering time QTLs in watermelon wild relative Citrullus amarus

**DOI:** 10.21203/rs.3.rs-7237247/v1

**Published:** 2025-08-28

**Authors:** Venkata Rao Ganaparthi, Patrick Wechter, Amnon Levi, Sandra Elaine Branham

**Affiliations:** Clemson University College of Agriculture Forestry and Life Sciences; Clemson University College of Agriculture Forestry and Life Sciences; USDA-ARS: USDA Agricultural Research Service; Clemson University

**Keywords:** Female flowering time, Fruiting time, Watermelon, QTL mapping, Citrullus amarus, Rootstock, Carolina strongback

## Abstract

Inbred lines of *Citrullus amarus*, a wild relative of cultivated watermelon, are widely used as rootstocks to control soil-borne diseases for watermelon (*Citrullus lanatus*) production. The most commonly used *C. amarus* rootstock, ‘Carolina strongback’ (Syngenta, Basel, Switzerland) flowers weeks later than commercial watermelon cultivars, which delays the onset of female flowering (DFF) of the scion, leading to an undesirable delay in fruit maturity and harvesting. Understanding the genetics of DFF in a *C. amarus* population will facilitate the development of rootstocks with the early flowering habits preferred for commercial production. A recombinant inbred line population (N = 129 lines) developed between *C. amarus* lines, USVL246-FR2 and USVL114, was evaluated in field trials in spring and fall of 2022 and 2023 for DFF and days to fruiting (DFT) after being transplanted into the field. The correlation between DFF and DFT is 0.92. Broad sense heritability of DFF and DFT were 0.23 and 0.31, respectively. Two QTLs influencing both DFF and DFT across the seasons and years were identified at 90.5 and 56.0 cM on chromosome 3 and together explained 39.67 percent variance of DFF. Two additional QTLs associated with DFF, were season-specific with a spring and a fall QTL on chromosome 10 and on the proximal end of chromosome 3, respectively. Genes coding for putative proteins involved in inducing anthesis, activation and regulation of FT proteins were identified in the 1.5 LOD interval of the stable major QTLs on chromosome 3.

## Key Message

Widely used *Citrullus amarus* rootstock ‘Carolina strongback’ delays female flowering of scion(s). Two stable QTLs influencing female flowering time and fruiting time across the seasons and years were identified on chromosome 3.

## Introduction

Determinants of crop success, such as biomass production and yield, are influenced by flowering time (FT) during the crop growth cycle. The flowering habit of a crop has been domesticated accordingly to maximize the monetary benefit of the crop (Cockram et al. 2007). Early FT is a desired trait in cereal and horticultural crops as it allows more time to accumulate biomass in floral parts to increase monetary benefits. In contrast, late FT is desired for biofuel or forage crops where the economical product is vegetative biomass. FT and fruit set are crucial fitness traits and influence the domestication of horticultural crops (Blümel et al. 2015). FT determines the time of fruit set and fruit maturity in watermelon (Jung and Müller, 2009). Early fruit maturity is a trait targeted by commercial breeding programs as it helps reduce input costs (Poland et al. 2009). In watermelon, early FT is particularly important as the demand and often purchase price for watermelon drastically increases for the US Independence Day holiday (July 4), where the fruit is commonly consumed as part of the celebrations (Garden clippings, 2024). If producers do not have the bulk of their fruit harvested by this date, their annual watermelon profits can decrease greatly. Thus, early flowering and fruiting lowers watermelon production risks and can increase grower profits.

*Citrullus lanatus* includes sweet, red fleshed edible watermelon, while *Citrullus amarus* includes inedible wild watermelons. Due to decades of preferential selection for flesh color, sweetness and yield, genetic variation for biotic and abiotic stress tolerance is narrow among the *C. lanatus* species (Levi et al. 2001). Conversely, *C. amarus* accessions exhibit high genetic diversity for biotic and abiotic stress tolerance and are widely used as parents in resistance introgression into cultivated watermelon (Branham et al. 2019a; Branham et al. 2019b; Ganaparthi et al., 2023, 2024; Rennberger et al., 2023). *C. amarus* accessions are also used as rootstocks to control soil-borne diseases such as *Fusarium* wilt, pythium root rot and root-knot nematodes (Keinath et al., 2019; Keinath and Hassell, 2014). There are no resistant watermelon cultivars/hybrids to *Fusarium* wilt caused by *Fusarium oxysporum* f. sp. *niveum* (*Fon*) race 2 or root-knot nematode or effective chemical management options, so the use of resistant rootstocks or abandonment of infested fields are the only management options (Waldo et al., 2023.). ‘Carolina strongback’, an inbred line developed from two *C. amarus* accessions, is used as a rootstock to prevent economic losses to *Fon* race 2 and root-knot nematode (Keinath and Hassell, 2014). Along with resistance to *Fon* race 2 and root-knot nematodes, rootstocks improve horticultural traits such as fruit yields and the flesh firmness of fruits produced by the *C. lanatus* scion (Devi et al. 2020). Rootstocks also influence the flowering time of the scion. Carolina strongback delays days to female flowering (DFF) and consequently delays fruit maturity and harvest. Therefore, understanding the genetics of flowering time in *C. amarus* is essential to develop improved rootstocks with an early flowering habit.

Flowering time is a vital and complex trait in all the major economic crops; hence, extensive research has been carried out to understand the genetic architecture and molecular pathways underlying flowering time in model systems such as *Arabidopsis thaliana* and maize (Blázquez et al. 2003; Shindo et al. 2005; Buckler et al. 2009). QTL mapping with *C. lanatus* populations and genome-wide association studies (GWAS) employing *C. amarus* accessions have been undertaken to understand the genetic architecture of FT in watermelons (McGregor et al. 2014; Gimode et al. 2020; Katuuramu et al. 2023). QTLs and QTNs influencing DFF were identified on chromosomes 2, 3, 4, 5, 8, 9 and 11. QTLs and QTNs with large allele effects, influencing DFF by five to ten days, were identified with both the QTL mapping and genome-wide association studies. However, QTL mapping of FT with a *C. amarus* bi-parental population has not reported, hindering marker development for early FT in wild watermelon. In addition, although watermelons are produced commercially in two planting seasons (spring and fall) in the US, South and Central America, existing studies have focused solely on FT in the spring season.

The objectives of our study were to identify stable large effect QTLs influencing DFF and days to fruit formation in a bi-parental *C. amarus* population, and identify season-specific FT QTLs.

## Materials and methods

### Plant materials and transplant growth conditions:

An F_8:9_ RIL population (N = 129 lines) segregating for DFF and DFT after transplantation was developed between USVL114 and USVL246-FR2 through single-seed descent, as described by Branham et al. (2019a). The parents are readily available from the USDA-National Plant Germplasm Repository. USVL114 produces female flowers and fruits thirteen days earlier (on average) than USVL246-FR2. For each field trial, five seeds of each RIL were hand sown into metro-mix 360 soilless media (Sun Gro Horticulture, Agawam, MA, USA) in 50-cell propagation trays. They were grown in a greenhouse with an average temperature of 25°C for four weeks. Seedlings were watered as needed and were fertilized with water-soluble fertilizer (NPK: 20:20:20) at a rate of 5g/L (Scotts, Marysville, OH, USA) on the 14th and 28th day after seeding.

### Field experiments:

Field studies were carried out at the Coastal Research and Education Center, Clemson University, Charleston, South Carolina. Loamy fine sand was the predominant soil type at the research station. Experiments were carried out in the spring and fall of 2022 and 2023. RILs, along with the parents, were seeded the last week of March and seedlings were hand transplanted into raised beds in the first week of April for the spring trials. Fall trials were seeded during the last week of May, and seedlings were transplanted in the first week of July. In all the experiments, three plants per genotype were transplanted onto the raised beds with 3-meter spacing between the genotypes and plants within a genotype were separated by 0.91 m. The experimental design for all four field trials was a randomized complete block with two replications with each replication treated as a block.

The fields were seeded with rye (*Secale cereale*) in the winter before each spring experiment. After all spring trial plots were harvested, the field was cleared and fall experiments were planted in the same field. The field was disked twice before transplantation for all the experiments, and 560 kg/ha of NPK:15-0-12.5 fertilizer was applied. Raised beds of width 0.9 m were shaped along the length of the field. Pre-emergent herbicides S-metolachlor and halosulfuron were sprayed on the raised beds and covered with black polyethylene mulch. Plots were hand-weeded on a weekly basis to reduce competition for crop growth. Insect and disease pressure were maintained below the threshold by spraying with chemicals according to the Southeastern US Vegetable Crop Handbook (2022). The vines of each plant were turned three times a week to maintain separation for data collection.

### Phenotypic data collection and statistical analysis:

In all the experiments, three plants per rep (for a total of six plants) of one hundred and twenty-nine RILs and the parents, were evaluated. The number of days from transplantation to the opening of the first female flower (DFF) and from the first appearance of fruit (DFT) was recorded for each plant.

Three sets of best linear unbiased estimates (BLUEs) and best linear unbiased predictions (BLUPs) were calculated for DFF and DFT of each genotype: 1) across spring trials, 2) across fall trials and 3) across all tests. The BLUEs was obtained using the following model implemented in lme4 package (Bates et al. 2015) of R (R Core Team 2022) with the following formula:

$$Y=g_{i}+r_{j}+y_{k}+g_{i}:y_{k}+e_{ij}$$

Where Y represents the BLUEs of each RIL, *g*_*i*_ is the fixed effect of the i^th^ RIL and *r*_*j*_ is the random effect of the j^th^ rep, *y*_*k*_ is the random effect of the k^th^ year/test, *g*_*i*_ : *y*_*k*_ is the interaction between i^th^ genotype and k^th^ year/test, and *e*_*ij*_ is the random error variance. The same model was used to obtain BLUPs, except that genotype was included as a random factor. Variances obtained from the BLUPs were used to estimate heritability. Broad-sense heritability of days to female flowering and fruiting for spring and fall seasons, and for across the seasons among the RILs in the study were calculated using the following formula (Piepho and Möhring 2007): $H^{2}=\frac{\sigma_{g}^{2}}{\sigma_{p}^{2}}$

$$\sigma_{p}^{2}=\sigma_{g}^{2}+\frac{\sigma_{gt}^{2}}{m}+\frac{\sigma^{2}}{rm}$$

Where $\sigma_{g}^{2}$ is the variance due to genotype, $\sigma_{p}^{2}$ is the phenotypic variance, $\sigma_{gt}^{2}$ is the variance of the genotype-by-test or year interaction, *m* is the number of test/year (experiments), and *r* is the number of replicates per trial. Mean DFF and DFT of genotypes for spring and fall seasons and across all tests were obtained with the ‘aggregate’ function implemented in R. Homogeneity of test and season variance was assessed with the Bartlett test (Bartlett, M. 1937) using the function ‘bartlett.test’ in R.

### QTL mapping:

A previously reported genetic map of the RIL population consisting of 2,174 polymorphic SNP markers developed from genotype-by-sequencing and KASP markers(Branham et al., 2019; Ganaparthi et al., 2024) was utilized for QTL mapping of DFF and DFT. Multiple QTL mapping with Haley-Knott regression (Haley and Knott 1992) was used to identify genomic regions associated with DFF and DFT in the RIL population. The optimum model with the highest penalized logarithm of the odds (LOD) score was determined with the stepwiseqtl function implemented in the R package ‘qtl’ (Broman and Sen 2009). The LOD significance threshold was established with 1,000 permutations using the scantwo function with penalties at *α* = 0.05. Interactions between QTLs were modeled with the option scan.pairs = T implemented in the scantwo function. LOD profile figures of all the linkage groups were generated with the scanone function, adding a single QTL at a time with the function addqtl to visualize forward model selection. The argument ‘expandtomarkers = T’ of the lodint function identified the markers flanking each significant QTL for the 1.5 LOD interval. USVL246-FR2 genome annotations(Wu et al. 2023) obtained from the CuGenDBv2 database were used to identify the candidate genes within the 1.5 LOD interval of each significant stable QTL and determine their putative protein function.

## Results

### Variation in days to flowering and fruiting among the RIL population:

**Figure 1:**
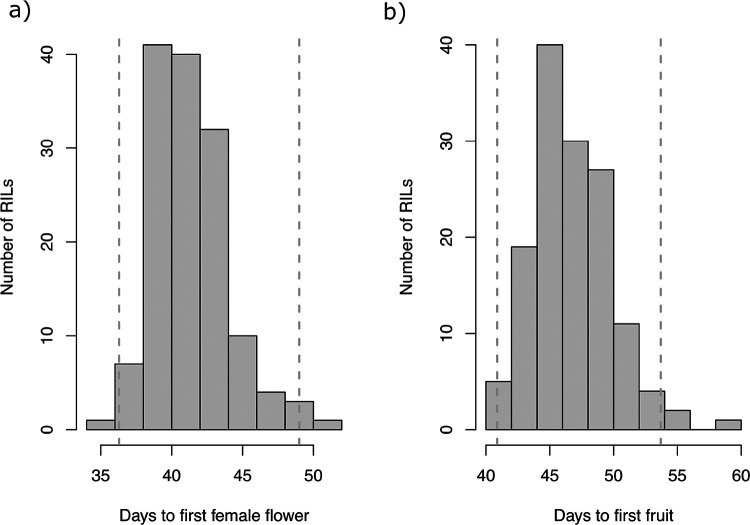
Histogram showing the distribution of a) days to appearance of first female flower, b) days to the appearance of first fruit after transplanted into the field across the spring and fall seasons of 2022 and 2023.

Across the years and seasons, mean DFF and DFT was 36.29 and 40.87 for USVL114 and 49.20 and 53.94 for USVL246-FR2, respectively (Supplementary table). Two RILs had lower mean DFF than USVL114 and one RIL had a higher mean DFF than USVL246-FR2 (Fig. 1), indicating transgressive segregation. The lowest number of DFF and DFT were recorded by RIL 200 (34.91 and 40.25, respectively), while the highest number of DFF and DFT were recorded by the RIL 215 (49.74 and 54.53, respectively). The variances of DFF and DFT were non-homogenous within year, test, season and replication (*P* value ranged from < 2e-16 to 0.025). Population line means of DFF ranged from 34.91 to 49.74 with a standard deviation of 2.47 (Table 1), while DFT ranged from 40.25 to 54.53. The population means for DFF and DFT did not vary by season (41 and 46 days, respectively for both the spring and fall). The range of DFF was 29.58 to 47.16 in the spring and 31.58 to 52.09 in the fall. The correlation between DFF and DFT was high both overall (0.92) and within each season (spring = 0.82 and fall = 0.90). Broad-sense heritability of DFF and DFT across the tests was 0.23 and 0.31, respectively. Broad-sense heritability was lower in the spring (DFF = 0.21 and DFT = 0.37) than the fall (DFF = 0.49 and DFT = 0.62).

### QTL mapping:

**Figure 2:**
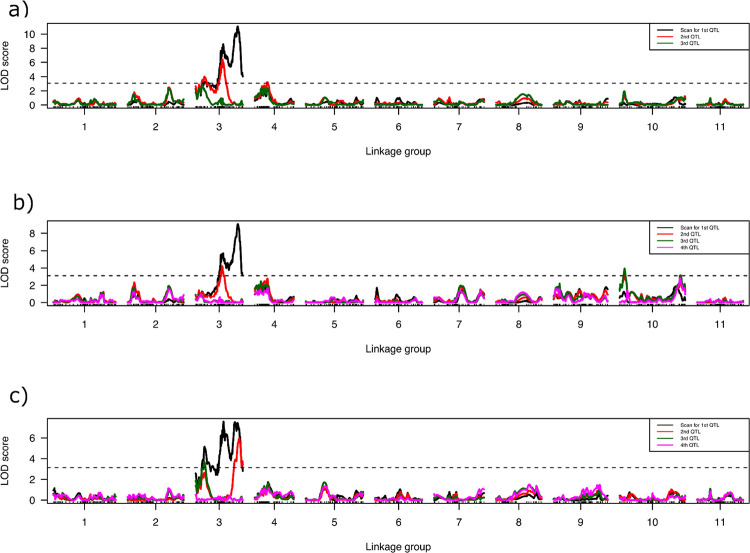
Logarithm of odds (LOD) scores for QTL associated with best linear unbiased estimates (BLUEs) obtained from the days to first female flower appearance after transplanted into the field a) Across the spring and fall seasons of 2022 and 2023, b) across the spring season of 2022 and 2023, c) across the fall season of 2022 and 2023. The horizontal dashed line indicates the genome-wide significance threshold.

QTL mapping was performed for six separate traits: BLUEs of DFF and DFT for the spring, fall and across all tests. The optimal multiple QTL mapping (MQM) model with the highest penalized LOD score was determined for each trait. A single major QTL was identified on chromosome 3 around 90 cM, consistently across the seasons and years. MQM of overall DFF BLUEs (i.e., across all four field trials) identified two QTLs on chromosome 3 (Fig. 2a). The QTL with the highest LOD score was identified on chromosome 3 at 90.5 cM and explained 22.69% of phenotypic variance (V_P_) with an additive effect of 1.15 (Table 3). A second QTL was identified on chromosome 3 at 56.0 cM with an LOD score of 7.89 and explained 16.98% of V_P_ with an additive effect of 0.99. No epistatic interaction between QTLs was identified. Similarly, MQM of BLUEs on DFT across the years and seasons identified two QTLs on chromosome 3. The position of identified QTLs on chromosome 3, their phenotypic variance and additive effects are similar to those identified for DFF (Table 3).

MQM identified three QTLs on chromosome 3 affecting the DFF of fall-transplanted watermelons. All three QTLs identified are on chromosome 3 at different positions i.e., 18.0 cM, 58.3 cM and 91.0 cM. Major QTL at 91.0 cM explained 15.90% of V_P_ with an additive effect of 1.14. Minor QTLs at 58.3 cM and 18.0 cM explained 9.10 and 7.24% V_P_, respectively. The same three QTLs were identified with MQM on BLUEs for DFT for the fall transplanted trials (Table 3).

Three QTLs influence DFF for the spring-planted fields and together explained 37.02% of V_P_. MQM identified two QTLs each on chromosome 3 and a single QTL on chromosome 10. Major QTL on chromosome 3 was identified with an LOD value of 11.12 at 90.00 cM and explained 19.22% V_P_. QTL on chromosome 10 was identified with a peak LOD score of 3.94 and 8.06% V_P_. No interaction was found among the QTLs.

Inbred lines with the BB (N = 58) allele at QTL3.1 flower approximately three days earlier, on average, than the lines with the AA (N = 69) allele. Similarly, On average, lines with the BB allele (N = 52) at QTL3.2 flower approximately two days earlier than the lines with AA allele (N = 76). Thirty inbred lines inherited AA alleles for both the QTLs (i.e., QTL3.1 and QTL 3.2) showed a mean DFF of 43.68, while the inbred lines with BB allele (N = 47) for the same QTLs showed a mean DFF of 39.38. On average, lines with BB alleles for the haplotype representing the two stable QTLs flowered 4.3 days earlier than the lines with AA alleles.

## Discussion

Flowering and fruiting time are two of the most important traits targeted in watermelon breeding programs (Wehner, 2008). Though wild *C. amarus* are not commercially grown, rootstocks of *C. amarus* are widely used in watermelon cultivation. Since the flowering and fruiting behaviour of watermelon rootstock influences the DFF, DFT and fruit maturity of commercially-grown scions, understanding the genetic nature of DFF and DFT with a *C. amarus* mapping population is crucial to develop rootstocks with an earlier flowering habit (Devi et al. 2020). *C. amarus* accessions are also employed in biotic and abiotic stress resistance breeding in watermelon breeding programs, and information about the genetic nature of DFF will increase the efficiency of introgression of targeted traits into a *C. lanatus* background.

The interval between the mean DFF and DFT among the RIL population is 4–5 days. Pollination, fertilization and fruit initiation in watermelon takes about 3–5 days. This study found a strong correlation between days to first female flower and days to fruiting among the mapping population, indicating low female flower abortion. DFF in watermelon has been studied previously with the USDA panel of *C. amarus* accessions (Katuuramu et al. 2023)d *lanatus* bi-parental population (McGregor et al., 2014). Mean DFF was highest among the *C. amarus* RIL population (41 days) employed in the current study, followed by the panel of *C. amarus* accessions (35 days)(Katuuramu et al. 2023) and the *C. lanatus* bi-parental population (26 days). The growing period of cultivated and wild watermelon ranges between 80–90 and 110–130 days, respectively (Guo et al., 2015). Hence, the time to DFF after transplanting is low among cultivated watermelons. Surprisingly, the average DFF is 35 days after transplantation among *C. amarus* accessions, while the average DFF among the current population is 41 DAT. Differences in populations and environmental conditions influence DFF (Turner et al. 2005; Buckler et al. 2009). Though both studies were carried out in Charleston, SC, USA,Katuuramu et al. (2023) transplanted seedlings into the field during the month of May, where mean temperatures are very suitable for vegetative to flowering phase transition (Jagadish et al. 2016). The current study was transplanted into the field at the beginning of April or July, when average temperatures and precipitation do not allow for the rapid transition from vegetative to flowering and fruiting phases.

The heritability of days to female flower in this study (H^2^ = 0.23) was much lower than the heritability reported for the whole panel of *C. amarus* accessions (0.75) (Katuuramu et al. 2023), however, is comparable to the heritability reported by McGregor et al. (2014) on a *C. lanatus* bi-parental RIL population (H^2^ = 0.23). Though McGregor et al. (2014) and the current study used RIL populations belonging to different species of *Citrullus*, both reported low broad-sense heritability for DFF. Low heritability in the current study could be due to the evaluation of the RIL population in two different climates, i.e., spring and fall seasons. However, the broad-sense heritability for each season separately was low to moderate (0.21 and 0.49, respectively) but not high (0.75), as reported by Katuuramu et al. (2023).

The QTL on the distal end of chromosome 3 @ 90.5 cM (30.61 Mb) was identified as the major QTL across the tests and seasons; hence is a stable QTL influencing DFF and explained 22.69% V_P_. Two other QTLs identified on chromosome 3 influenced flowering behaviour in fall transplanted tests at 18.0 and 58.3 cM (5.34 and 20.97 Mb). McGregor et al. (2014) identified three QTLs on chromosome 3 that were associated with flowering time in a *C. lanatus* RIL population. The major QTL beginning of chromosome 3 at 17 Mb explained 44.7% V_P_ and showed an additive effect of 2.5 DFF (McGregor et al. 2014). Further fine mapping identified a single SNP on chromosome 3 at 15.4 Mb with an allele effect of 11 DFF (Gimode et al. 2020). The major QTL identified on chromosome 3 in this study was also associated with flowering time across *C. amarus* accessions but had an additive effect of only − 1.15 DFF. This allele effect size discrepancy among the cultivated and wild watermelon can be explained by artificial selection for large-effect QTLs (Dittmar et al. 2016). *C. amarus* is a population consists of naturally evolved wild watermelons. On the contrary, edible red-fleshed watermelon species, *C. lanatus* are subjected to artificial selection for agronomical and horticultural traits during and after domestication. Early flowering habit is one such agronomical trait targeted during domestication and by the commercial breeding programs, which might had let to the selection for a few large-effect QTLs and many small-effect QTLs. Such large effect QTLs are absent among parents employed to develop the mapping population, thus were not found in the current study. Traits important for domestication and being controlled by a single large-effect QTL and many small effect QTLs were identified among cucurbits (Tzuri et al. 2015).

A total of 215 genes were located within the 1.5 LOD intervals of the two stable QTLs identified on Chromosome 3 at 56.0 and 90.5 cM. The strongest putative candidate genes within these ranges encode proteins with functional roles in the transition from the vegetative to reproductive phase. Candidate gene CaUCo3G059780 (chromosome 3 @ 56 cM) encodes a Flowering Locus T (FT) protein, which are well known flowering time regulated by the photoperiod and vernalization, and act as the primary flowering signals for floral induction in Arabidopsis, Rice and Barley (Faure et al. 2007; Tsuji and Sato 2024). Gibberellin receptors mediate gibberellin pathways leading to the activation of FT, which in turn initiates flowering (Mutasa-Göttgens and Hedden, 2009). In addition, gibberellin signalling is essential for the normal development of floral structures (Achard et al. 2004). A gene (CaUC03G065560) predicted to encode the gibberellin receptor GID1C was identified in the QTL interval on Chromosome 3 at 90.5 cM (31.16 Mb).

Rootstocks with early flowering habit and *Fusarium* wilt race 2 resistance is highly desirable for commercial watermelon production. *Fusarium* wilt race 2 resistance and DFF among the bi-parental population employed in the current study were not significantly correlated (*P*-value = 0.27, rho = −0.09). The five RILs with the lowest DFF are all susceptible to *Fusarium* wilt race 2 (Ganaparthi et al. 2024). Therefore, the development of rootstocks with resistance to *Fusarium* wilt race 2, root-knot nematode and early flowering habit may require large population sizes with extensive phenotyping to combine all the desired traits into an elite rootstock line.

This study identified two stable QTLs influencing DFF across the years and seasons. QTLs identified among the *C. amarus* population showed small additive effects indicating the quantitative nature of the trait. Resistance to *Fusarium* wilt race 2 and root-knot nematode are also quantitatively inherited (Waldo et al. 2023; Ganaparthi et al. 2024), genomic selection could be an optimal tool to develop rootstocks with both resistances and an earlier flowering habit.

## Supplementary Material

Supplementary Files

This is a list of supplementary files associated with this preprint. Click to download.

- supplementarytable.xlsx

## Figures and Tables

**Figure 3:**
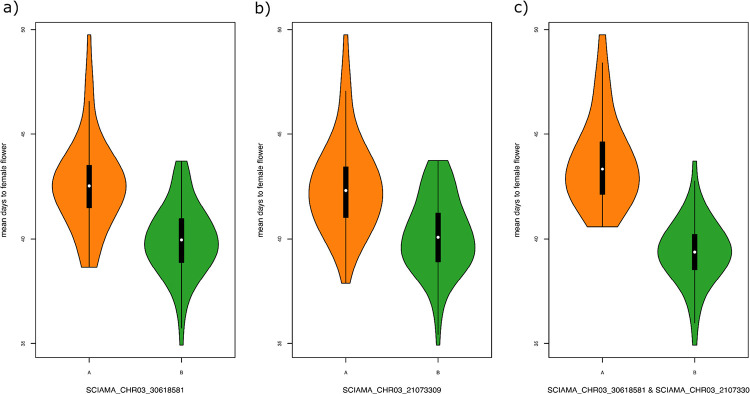
Effect plots showing the mean and standard errors for best linear unbiased estimates (BLUEs) of days to the appearance of first female flower for genotypic class: AA homozygous resistant parent alleles, BB homozygous susceptible parent alleles. Panels depict the genotypes of the SNP with highest LOD scores for each quantitative trait loci identified across the field trials: a) SNP at the QTL on Chromosome 3 at 90.50 cM; b) SNP at the QTL on chromosome 3 at 56.00 cM.

**Figure 4:**
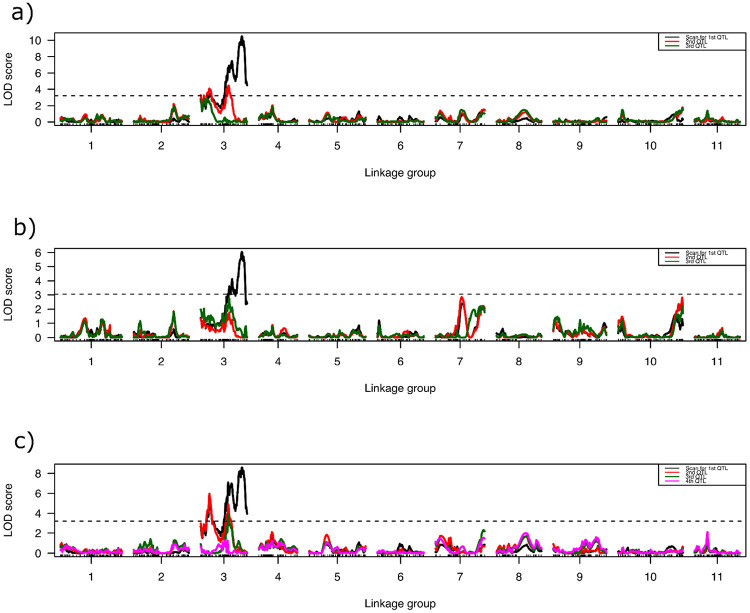
Logarithm of odds (LOD) scores for QTL associated with best linear unbiased estimates (BLUEs) obtained from the days to first fruit appearance after transplanted into the field a) Across the spring and fall seasons of 2022 and 2023, b) across the spring season of 2022 and 2023, c) across the fall season of 2022 and 2023. The horizontal dashed line indicates the genome-wide significance threshold.

**Table 1:** Summary statistics for days to female flowering and fruiting after transplanting into the field across 129 RILs developed between USVL246-FR2 and USVL114 across four field seasons at the Coastal Research and Education Center in Charleston, South Carolina.

Trait	Mean ± SD	Min	Max
Days to female flowering (Across the trials)	41.13 ± 2.47	34.91	49.75
Days to fruiting (Across the trials)	46.58 ± 2.71	40.25	54.53
Days to female flowering (Spring trials)	41.07 ± 2.47	29.5	47.16
Days to fruiting (Spring trials)	46.71 ± 2.68	34.91	52.58
Days to female flowering (Fall trials)	41.13 ± 3.01	31.58	52.09
Days to fruiting (Fall trials)	46.39 ± 3.42	39.5	56.76

**Table 2:** Correlations among female flowering and fruiting after transplanting into the field across 129 RILs developed between USVL246-FR2 and USVL114 across four field seasons at the Coastal Research and Education Center in Charleston, South Carolina.

Traits	fruit	Spring female flower	Spring fruit	Fall female flower	Fall fruit
Female flower	0.92	0.85	0.72	0.9	0.87
Fruit		0.79	0.84	0.82	0.91
Spring female flower			0.82	0.56	0.6
Spring fruit				0.49	0.54
Fall female flower					0.9

**Table 3:** Quantitative trait loci (QTL) associated with Days to female flowering and Days to fruiting after transplantation across the 2022 and 2023 field seasons, and across the spring and fall seasons of 2022 and 2023.

Trait	Season	Chr	Position (cM)	LOD	Peak LOD position (Mb)	% V_p_	Additive effect
DFF	Overall	3	90.5	10.10	30.61	22.69	−1.15
DFF	Overall	3	56.0	7.88	21.07	16.98	−0.99
DFT	Overall	3	91.0	9.46	30.61	22.57	−1.29
DFT	Overall	3	59.5	6.27	23.13	14.10	−1.01
DFF	Spring	3	90.0	11.12	30.61	19.22	−1.10
DFF	Spring	3	54.7	6.18	20.12	9.74	−0.78
DFF	Spring	10	10.3	3.94	6.44	8.06	−0.70
DFT	Spring	3	91.5	6.70	30.61	19.57	−1.19
DFF	Fall	3	91.0	7.27	30.61	15.89	−1.14
DFF	Fall	3	58.3	4.34	22.71	9.39	−0.89
DFF	Fall	3	18.0	3.73	5.34	7.62	−0.81
DFT	Fall	3	91.5	9.09	30.61	19.66	−1.51
DFT	Fall	3	58.5	4.20	22.71	8.29	−1.03
DFT	Fall	3	18.0	3.98	5.34	7.83	−0.99

## Data Availability

The datasets generated during and/or analysed during the current study are included in the supplementary tables.
